# Unmasking Carpal Tunnel Syndrome by High-Resolution Ultrasonography in Misdiagnosed Pure Neuritic Leprosy: A Case Report

**DOI:** 10.7759/cureus.65578

**Published:** 2024-07-28

**Authors:** Kalyan Dalave, Priyanka Patil, Tamanna Raman, Urvashi Agarwal

**Affiliations:** 1 Dermatology, Dr. D. Y. Patil Medical College, Hospital and Research Centre, Dr. D. Y. Patil Vidyapeeth, Pune, IND; 2 Radiodiagnosis, Dr. D. Y. Patil Medical College, Hospital and Research Centre, Dr. D. Y. Patil Vidyapeeth, Pune, IND

**Keywords:** hansen's disease, high-resolution ultrasonography (hrus), carpal tunnel syndome, high-resolution ultrasonography, pure neuritic leprosy, misdiagnosed leprosy

## Abstract

We present a case of a woman in her 70s who was initially diagnosed with pure neuritic leprosy due to bilateral hand numbness and tingling with associated muscle weakness. However, after undergoing high-resolution ultrasonography (HRUS), it was found that she had bilateral carpal tunnel syndrome (CTS). This case highlights the importance of considering other possible causes of peripheral neuropathy, such as CTS, in patients with suspected Hansen's disease. It also establishes the role of HRUS in the prompt diagnosis of CTS. Appropriate treatment of CTS can prevent unnecessary and potentially harmful treatment for Hansen's disease.

## Introduction

Leprosy is a chronic infectious disease affecting the skin, peripheral nerves, and mucous membranes, leading to a wide spectrum of clinical manifestations. Pure neuritic leprosy (PNL) is a form of leprosy commonly seen in India and Brazil. It only affects the peripheral nerves, without any visible cutaneous lesions [1]. The presentation can be in the form of tingling and numbness in affected nerve distribution, muscle weakness, and visible deformity. The clinical presentation of PNL can mimic other forms of peripheral neuropathy, making it challenging to diagnose. Carpal tunnel syndrome (CTS) is a common form of peripheral neuropathy caused by the involvement of the median nerve. It can also present with numbness and tingling in the hands [2]. We present a case of bilateral CTS misdiagnosed as PNL where high-resolution ultrasonography (HRUS) played a major role in reaching to the correct diagnosis.

## Case presentation

**Figure 1:**
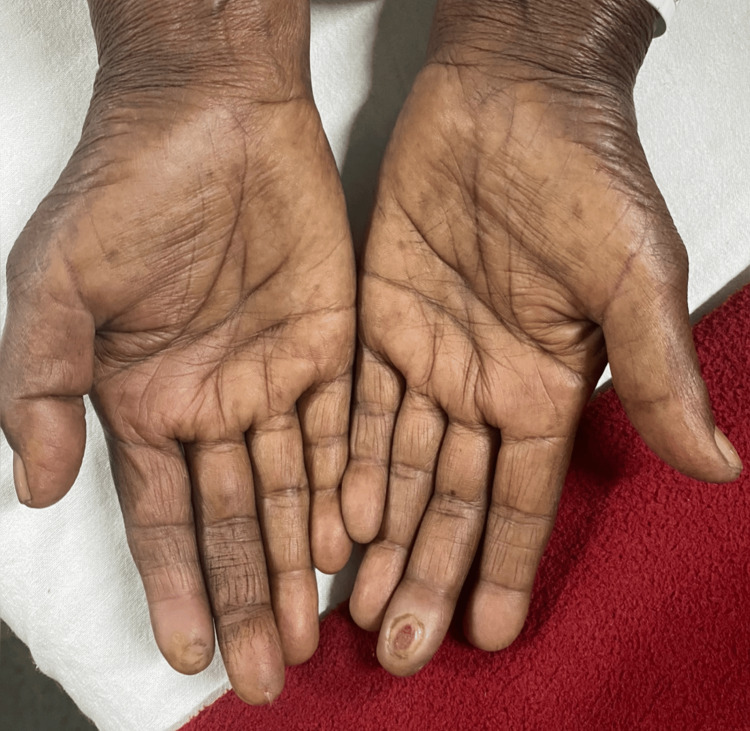
Clinical image depicting an ulcer over the tip of the left middle finger.

A 72-year-old female presented to the outpatient department with a complaint of tingling and numbness over both hands for the past six months. She was hypertensive and diabetic and was taking telmisartan 40 mg and metformin 500 mg twice a day for the last 30 years. The patient was already on multidrug therapy (MDT) including once a month supervised dose of rifampicin 600 mg, clofazimine 300 mg, and dapsone 100 mg and once daily dose of clofazimine 50 mg and dapsone 100 mg when she presented to us. She had been diagnosed with PNL for the same complaints by a clinical practitioner and was started on MDT for the past four months. The patient had no relief in symptoms. On asking, she gave a history of intermittent low-grade fever episodes and arthralgia. On examination, the patient had no visible skin lesions suggestive of leprosy, except for an ulcer seen on the tip of the left middle finger (Figure 1.)

She had numbness and tingling in the distribution of the median nerve bilaterally with decreased pin-prick sensation and reduced hot and cold sensation in the same distribution. On nerve palpation, only the right ulnar nerve was slightly thickened. There was muscle weakness and partial clawing. No swelling or tenderness was present around the wrist joint. A slit skin smear was performed and was negative for lepra bacilli. All the examination findings were pointing toward the initial diagnosis of PNL.

**Figure 2:**
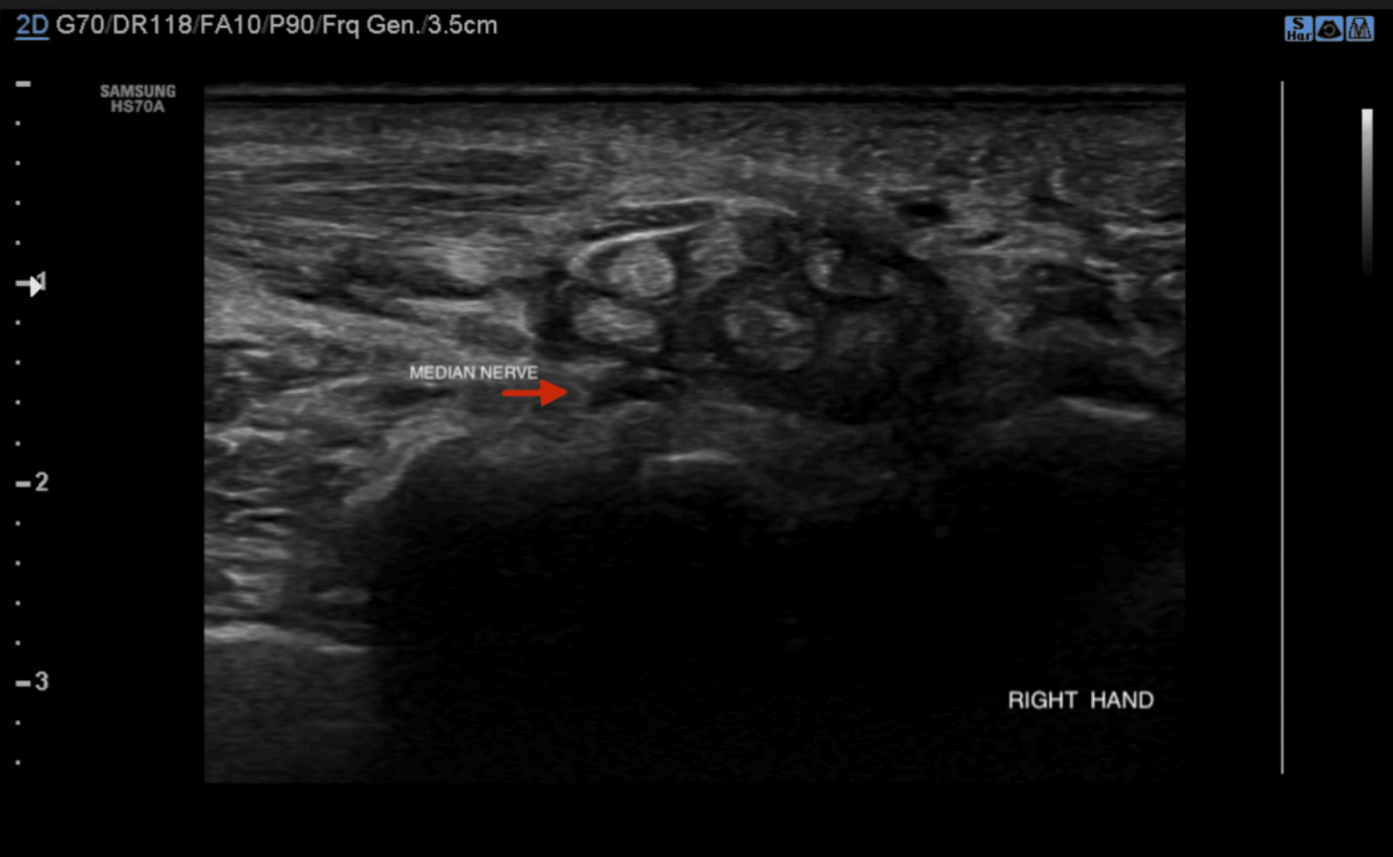
High-resolution ultrasonography of the right median nerve at the wrist High-resolution ultrasonography of the right median nerve showing median nerve in relation to flexor retinaculum. Loss of fascicular architecture and echotexture of the nerve with flattening of the nerve can be observed with palmar bowing of the flexor retinaculum. Flexor and extensor tendons appear bulky and echogenic with surrounding fluid, indicating the likelihood of tendinitis.

**Figure 3:**
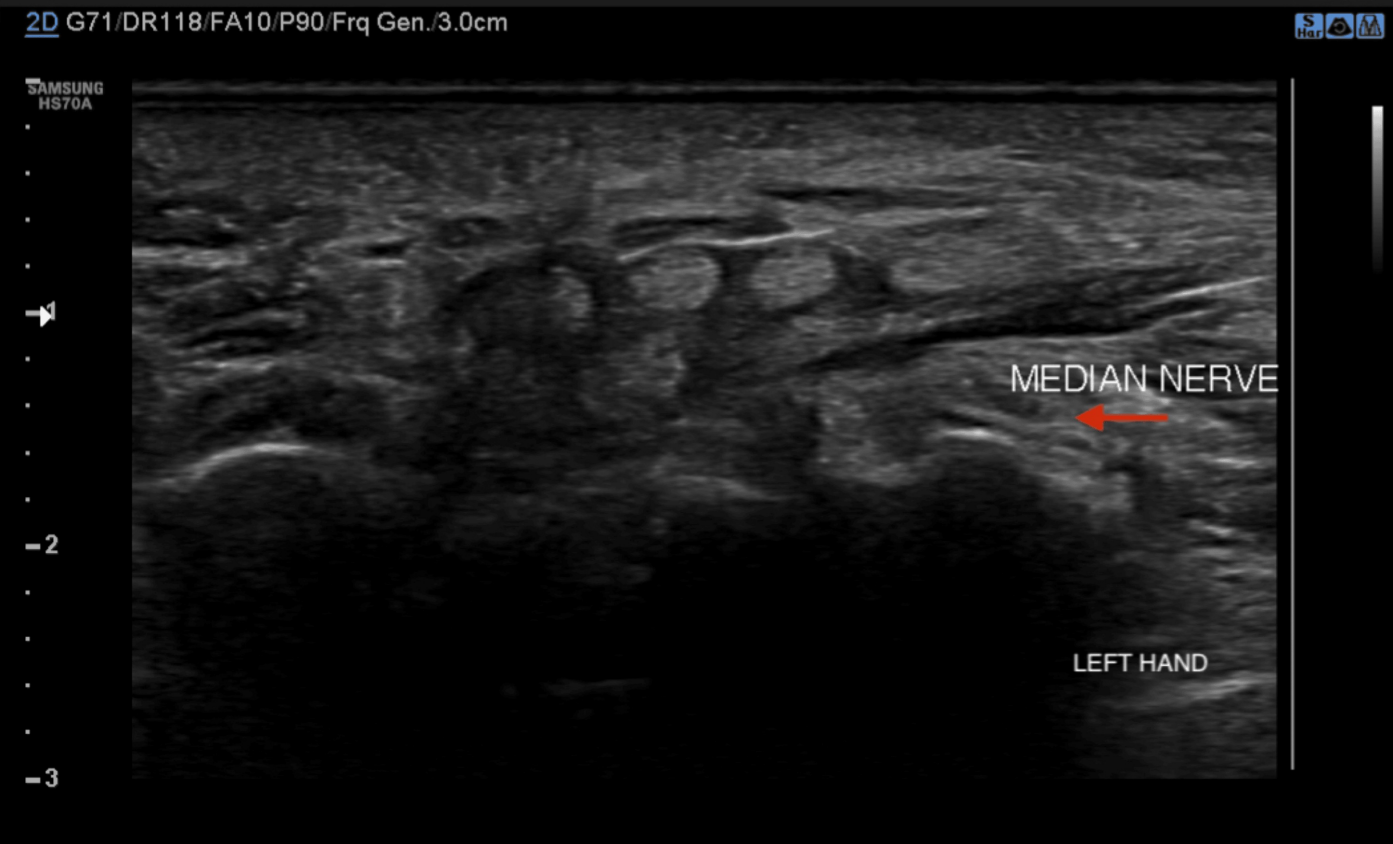
High-resolution ultrasonography of the left median nerve at the wrist High-resolution ultrasonography of the left median nerve showing the median nerve in relation to the flexor retinaculum. Loss of fascicular architecture and echotexture of the nerve with flattening of the nerve is observed with palmar bowing of the flexor retinaculum. Flexor and extensor tendons appear bulky and echogenic with surrounding fluid, indicating the likelihood of tendinitis.

The patient was referred to neurology in view of nerve conduction velocity (NCV) studies, which were suggestive of axonal sensory-motor neuropathy involving the bilateral median nerves. Since the patient was already on MDT and did not show any signs of lepra reaction, she was advised nerve biopsy to come to a definite diagnosis. The patient refused for biopsy as it was an invasive procedure. High-resolution ultrasonography with color Doppler was done to examine the ulnar and median nerves, which showed no significant increase in the cross-sectional area (CSA) for the ulnar nerve. However, the presence of bilateral median nerve entrapment at the wrist, flexor, and extensor tendinitis with increased subcutaneous edema in the surrounding area was observed; these findings were consistent with CTS. The CSA was noted to be 10 mm^2^, with distal flattening of the median nerves and palmar bowing of the flexor retinaculum (Figures 2, 3.)

The diagnosis of CTS was made, and the patient was advised to stop MDT and was referred to the orthopedic department for further management. The patient was treated conservatively, including wrist splints and pain management. The patient's symptoms showed significant improvement in the form of reduced tingling and muscle weakness.

## Discussion

PNL is a form of leprosy affecting peripheral nerves without the presence of any cutaneous lesions. Therefore, a diagnostic challenge arises as its presentations may mimic closely to peripheral neuropathies. PNL is more prevalent in the Indian subcontinent, and although its prevalence is low (5-18% of all leprosy diagnoses), it may be underdiagnosed because of a lack of readily available, non-invasive, definitive diagnostic tools available for detecting this type of leprosy [1].

In developing countries like India, leprosy is the most common cause of peripheral neuropathy [3] and hence considering the provisional diagnosis of PNL was justified in our case. Although there were no skin lesions suggestive of leprosy, the presence of motor and sensory weakness with partial clawing of the hand in the patient raised the possibility of PNL at first. The history of intermittent fever episodes, nerve thickening, and arthralgia was pointing toward a lepra reaction. The patient had grade one thickening of her right ulnar nerve without any tenderness or nodularity. A marginally thickened, non-tender, right ulnar nerve is a normal finding in a right-handed person [4]. The non-response of the patient to MDT was also pointing toward considering a different diagnosis.

The carpal tunnel region is bounded by the flexor retinaculum ventrally and the carpal bones dorsally. The median nerve courses below the flexor retinaculum ligament, ventrally and parallel to the flexor tendons. CTS occurs when there is a restriction of this space [2]. Diagnosing it early prevents permanent nerve damage and functional sequelae. The diagnosis of CTS relies on clinical signs and symptoms like the Tinel sign (where tapping the median nerve causes tingling) and the Phalen sign (where wrist flexion produces tingling), in addition to nerve conduction studies [2]. The incidence of CTS is higher in females and more common to occur in the dominant hand [5].

Buchberger et al. demonstrated in their study that certain sonographic signs can assist in diagnosing CTS. These include an increase in the cross-sectional area (CSA) of the median nerve to 10 mm² in the proximal or middle segments, a distal flattening ratio greater than three, and a displacement of the flexor retinaculum exceeding 4 mm [2]. Another study reported that among these three findings, the most common were diffuse localized swelling and increased flattening of the median nerve [6]. A less frequently seen sign was the increased bowing of the flexor retinaculum. Median nerve compression presents as a classic triad of proximal/middle nerve swelling, distal nerve flattening, and palmar bowing of the flexor retinaculum on HRUS [6]. There are several benefits to using ultrasonography for nerve assessment, including low cost, reduced study time, non-invasive procedures, and dependable results. This method enables dynamic imaging and evaluation of various parameters of the median nerve, such as size, vascularity, mobility, anatomical differences, and surrounding tissues.

Neves et al. in their study on misdiagnosed leprosy cases found that the misdiagnoses were discovered after patients presented with adverse effects to MDT or worsening of symptoms. The diseases most frequently reported as conclusive diagnoses in the study were osteoarticular and connective tissue disorders, skin and subcutaneous tissue diseases, and nervous system disorders like amyotrophic lateral sclerosis, neuropathies, and psychosomatic/depressive disorders [7].

Our case highlights the importance of considering other possible causes of peripheral neuropathy, like CTS, in patients with suspected PNL. NCS is commonly used in diagnosing CTS and can help differentiate it from other forms of peripheral neuropathy but was not definitive in our case [4]. NCS is anyways expensive and time-consuming. HRUS hence becomes a superior choice in correctly locating the nerve damage and establishing a diagnosis. HRUS helps in early detection of leprous neuropathy and differentiating it from other peripheral neuropathies.

## Conclusions

This case highlights the diagnostic complexities between PNL and other causes of peripheral neuropathies like CTS. Initially diagnosed with PNL, our patient's symptoms persisted despite treatment with MDT, prompting further investigation. HRUS revealed bilateral CTS, a diagnosis overlooked due to overlapping symptoms with PNL. HRUS proved crucial in guiding appropriate management and avoiding prolonged unnecessary treatment with leprosy drugs. This underscores HRUS's role as a non-invasive, cost-effective tool for accurately diagnosing peripheral nerve disorders, emphasizing the need for vigilant differential diagnosis in peripheral neuropathic conditions like leprosy.
